# Human Milk Microbiota Profile Affected by Prematurity in Argentinian Lactating Women

**DOI:** 10.3390/microorganisms11041090

**Published:** 2023-04-21

**Authors:** Sofía Oddi, Anastasia Mantziari, Paula Huber, Ana Binetti, Seppo Salminen, Maria Carmen Collado, Gabriel Vinderola

**Affiliations:** 1 Instituto de Lactología Industrial (INLAIN, UNL-CONICET), Facultad de Ingeniería Química, Universidad Nacional del Litoral, Santa Fe 3000, Argentina; 2 Functional Foods Forum, Faculty of Medicine, University of Turku, 20520 Turku, Finland; 3 Laboratorio de Plancton, Instituto Nacional de Limnología (INALI, UNL-CONICET), Universidad Nacional del Litoral, Santa Fe 3000, Argentina; 4 Departamento de Hydrobiologia, Universidade Federal de São Carlos (UFSCar), Rodovia Washington Luiz, São Carlos 13565-905, SP, Brazil; 5 Department of Biotechnology, Institute of Agrochemistry and Food Technology, Spanish National Research Council (IATA-CSIC), 46980 Valencia, Spain

**Keywords:** human milk, microbiota, full-term, pre-term, *Rothia*, alpha diversity

## Abstract

To study (16S rRNA-sequencing) the impact of gestational and corrected ages on the microbiota profile of human milk (HM) of mothers that delivered full-term and pre-term children, HM samples were obtained and classified according to the gestational age as group T (full-term births ≥37 weeks), and group P (pre-term births <37 weeks). Group P was longitudinally followed, and the samples were collected at the full-term corrected gestational age: when the chronological age plus the gestational age were ≥37 weeks (PT group). The HM microbiota composition differed depending on the gestational age (T vs. P). Group T had lower levels of *Staphylococcus* and higher levels of *Rothia* and *Streptococcus*, as compared to group P. The alpha Simpson diversity value was higher in group T than in P, whereas no differences were found between groups T and PT, suggesting a microbial evolution of the composition of group P towards group T over chronological age. Full-term delivery was associated with a greater diversity of microbes in HM. The microbial composition of pre-term HM, at the corrected age, did not show significant differences, as compared to the samples obtained from the full-term group, suggesting that it would be appropriate to consider the corrected age in terms of the composition and the diversity of the milk in future studies.

## 1. Introduction

Human milk (HM) is the gold standard for infant nutrition due to its complex and dynamic composition that protects a newborn against infections and intestinal inflammation by promoting the maturation of the intestinal barrier and the immune system [1]. The HM bioactive components and immune factors comprise immunoglobulins, lactoferrin, lysozyme, antimicrobial peptides, growth factors, white blood cells, microRNAs, human milk oligosaccharides (HMOs), and metabolites, among others [1]. In addition, HM is a unique source of commensal, mutualistic, and putative probiotic bacteria, such as *Bifidobacterium* and *Lactobacillus* [2]. Moreover, breastfeeding is recommended to prevent complications associated with pre-term birth, as it has been demonstrated to reduce the risk of neonatal sepsis, necrotizing enterocolitis, bronchopulmonary dysplasia [3], and other morbidities in pre-term neonates.

Perinatal factors shape the HM bioactive components, including the microbial profile [4]. In terms of nutrition status, milk from obese people presented low diversity, high levels of Staphylococcus, and low levels of Bifidobacteria, as compared to normal weight people. Maternal geographical location also seemed to modulate milk microbiota. For example, Chinese people have a higher level of *Streptococcus* in their milk than people from South Africa, Finland, and Spain. On the other hand, Spanish people present higher levels of *Cutibacterium* and *Pseudomonas* [5]. A recent study in Dubai showed differences between milk from Emirati and UAE-expatriated people’s HMO composition and specific microorganisms [6]. These previous reports have revealed the contribution of geographical location and lifestyle to HM composition and the importance of this study as the first one of its type in an Argentinian population.

Furthermore, it has been demonstrated that HM composition differed between people who give birth prematurely and those with full-term deliveries [7,8,9]. Specific HM components, such as insulin [10], lactose, fat, and the total energy content of milk, were not affected by gestational age. Nevertheless, the content of specific individual proteins, specific polar lipids [11], and cytokines [12] were significantly altered by both the gestational and chronological ages [13]. The nutritional composition of pre-term HM has been widely studied; however, its microbial composition has not yet been fully defined [1].

The concept of corrected age was recommended when following pre-term infants’ development for up to three years because their development would be delayed, as compared to full-term infants [14]. The corrected age was defined as the chronological age minus the number of weeks the infant was born early (prior to 40 weeks, gestational age) [15]. Although it is generally accepted that the corrected age is related to growth parameters, cognitive scores, and neurodevelopment, its impact on HM microbiota was unknown. As the overall HM composition (nutrients and bioactive components) strongly depends on the type of birth, pre-term or full-term, a similar effect was expected for their respective microbiota profiles. Therefore, this work aimed to characterize the HM microbiota of healthy Argentinian women who delivered at different gestational ages and to study the changes in milk microbiota according to the chronological age of infants delivered pre-term.

## 2. Materials and Methods

### 2.1. Subjects, Design, and Sample Collection

Twenty-four healthy breastfeeding mothers from Santa Fe, Argentina, were recruited. Half of the people had full-term deliveries and donated one sample of HM. The rest had pre-term deliveries and donated two samples of HM. The groups of the sample set were as follows: group T (HM samples from people who had full-term births ≥37 weeks of gestation, n = 12), and group P (HM samples from people that had pre-term births <37 weeks of gestation, n = 12), sampled during the first postpartum days. In addition, the PT group (n = 12) consisted of the people in group P, but a second sample was collected between 0 and 4 weeks of corrected age. Mothers from group T constituted the full-term birth group of spontaneous donating people in Santa Fe city, Argentina. Mothers from groups P and PT were recruited from the public (J. B. Iturraspe) and private (San Gerónimo) hospitals in the city of Santa Fe, Argentina.

Written informed consent was obtained from all participants. Approval from the Ethical Committee of the Province of Santa Fe (number 640/2017) was obtained. For the HM collection, breast skin was cleaned with soap and water, and the first 500–600 μL of HM were discarded. Less than 5 mL were collected from pre-term people, while 5–10 mL were collected from the full-term group. HM was collected in sterile containers, refrigerated, and processed in the laboratory within less than two hours of collection.

### 2.2. DNA Extraction and 16S rRNA Amplicon Sequencing

HM samples (1.5 mL) were centrifuged (20,000× *g*, 20 min, 4 °C) to remove fat and washed twice with 500 µL saline solution. For total DNA extraction, MasterPure™ Complete DNA and RNA Purification Kit (Epicentre, Madison, WI, USA) was used. DNA concentrations were measured using a Qubit 2.0 fluorometer (Life Technology, Carlsbad, CA, USA) and normalized to 5 ng μL^−1^ for 16S rRNA gene (V3–V4 regions) sequencing using Nextera XT Index Kit. Amplicons were reviewed with a Bioanalyzer DNA 1000 chip, and libraries were sequenced using a 2 × 300 bp paired-end run (MiSeq Reagent kit v3) on a MiSeq–Illumina platform (FISABIO sequencing service, Valencia, Spain).

### 2.3. Data Analysis

Raw fastq sequences were processed using a modified version of the pipeline proposed by Logares [16], described in the Supplementary Methods. Operational taxonomic units (OTUs) were defined with no clustering (zero-radius OTUs (zOTUs)) using the UNOISE2 algorithm [17]. The sequencing yielded a total of 2,299,797 reads (67,641 ± 40,237) in 34 samples. In addition, the analysis did not consider the sample PT1 with insufficient reads (<10,000), as compared to the rest of the samples (12,437 to 129,871 reads). Sample P6 was not considered because it showed an irregular distribution and higher abundance in most OTUs, as compared to other samples. The zOTU rarefaction curves were constructed, using vegan v.2.5 to evaluate whether the richness had been saturated. The rarefaction of each sample was performed to all the analyses, including the taxonomic diversity and richness determination. The taxonomic diversity and richness of the communities were estimated with the Simpson, Simpson Inverse [18], and Chao1 [19] indices, using vegan v.2.5. In addition, R software 4.1.3 [20] was used for the NMDS plot. The NMDS plot showed the group distribution according to a dissimilarity matrix. 

### 2.4. Statistical Analysis

PERMANOVA (9999 permutations) was performed on Bray–Curtis triangular matrices to examine differences in the composition of bacterial zOTUs in groups P, PT, and T using PAST software (Øyvind Hammer, Natural History Museum, University of Oslo) [21]. Then, a SIMPER analysis was performed to determine the OTUs that contributed to the differences between groups. Independent groups, pairs P vs. T and PT vs. T, were compared using the Student’s *t*-test and Mann–Whitney test, according to the data distribution. Instead, the pair P vs. PT was analyzed with a paired *t*-test and Wilcoxon Rank Sum Test, according to the data distribution. The Ryan–Joiner test for normality was performed to analyze the normal distribution of the relative abundance at phylum, family, and genus levels. Significant differences were considered at *p* < 0.05. The PAST software (Øyvind Hammer, Natural History Museum, University of Oslo) [21] was used for all the analyses.

In addition, the association of alpha diversity and taxa abundance at the phylum level, and specific maternal and infant factors were assessed by linear regression. The independent variables were delivery mode, administration of antibiotics, and prenatal corticoids. The dependent variables considered were phyla and alpha diversity indices. The continuous variables with no normal distribution were center log-ratio transformed following zero-replacement [22]. After the individual linear regressions using SSPS software, the β coefficient at *p* < 0.05 was used in the ClustVis web tool to visualize the heatmap (Table S1, Supplementary Materials) [23]. Then, Spearman’s rank correlation was carried out, considering the taxa abundance at phylum and genera levels; alpha diversity indices; and gestational, chronological, and corrected ages by SPSS software. It included *Staphylococcus* and *Streptococcus*, and less abundant genera, such as *Bifidobacterium*, *Enterobacter*, *Pseudomonas*, *Ralstonia*, and *Rothia genus* (>1% abundance and showing significant differences among groups). ClustVis web tool was used to generate the heatmap [23].

## 3. Results

### 3.1. Clinical Data Profile and Human Milk Microbiota Profile

No differences in clinical data were observed between pre-term and full-term groups, except for the gestational age and weight (Table S2, Supplementary Materials).

**Figure 1:**
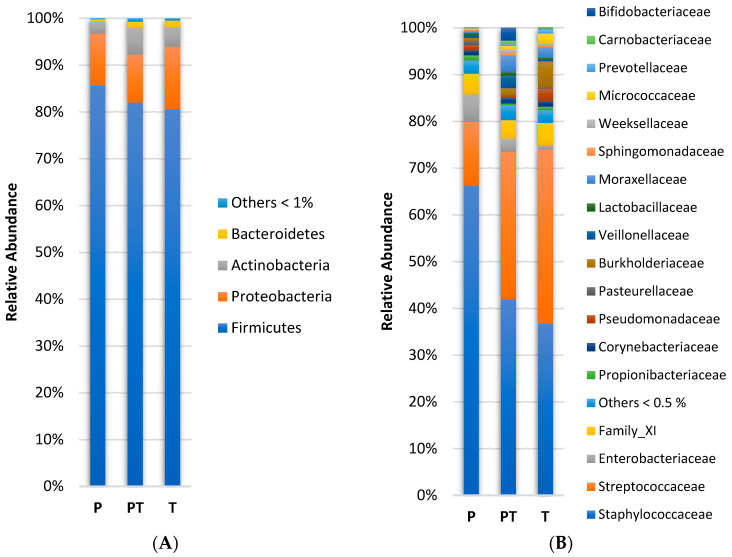

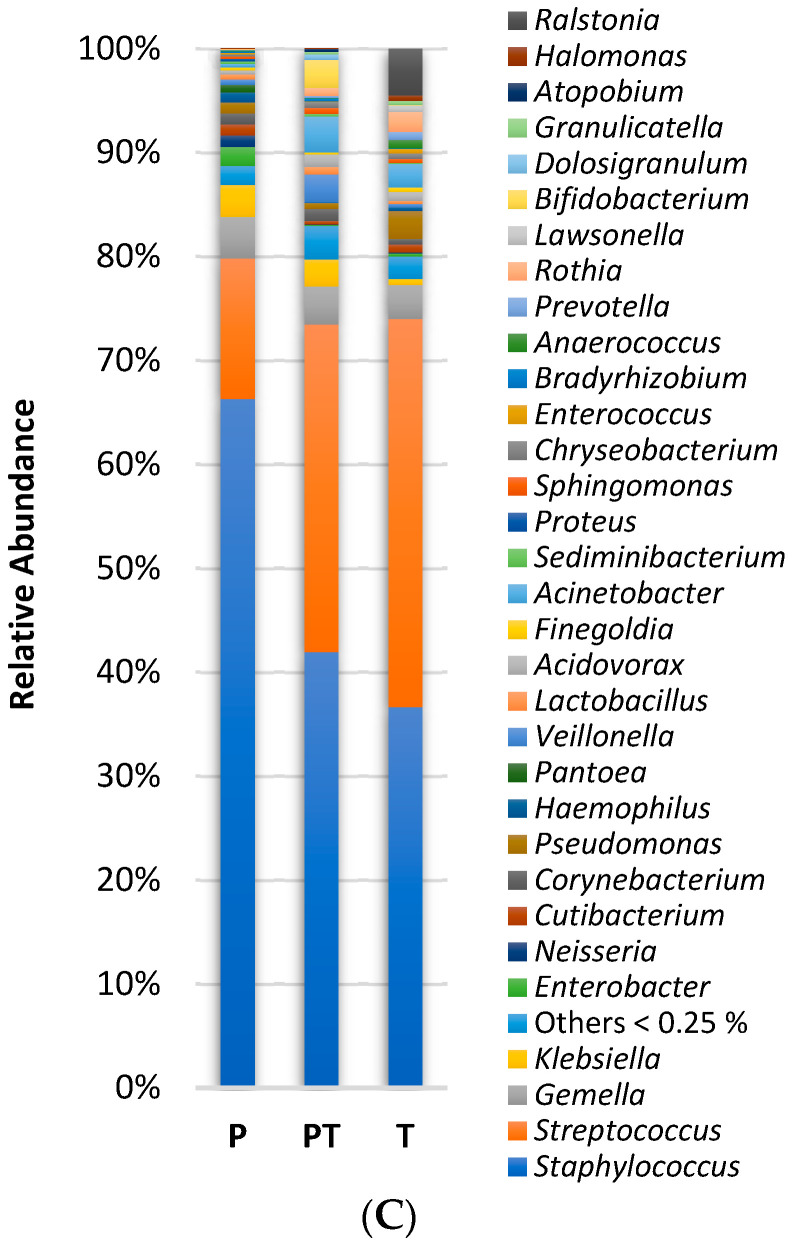
Relative abundance in human milk samples by 16S rRNA gene-sequencing at the phylum (**A**); at the family (**B**); and at the genus levels (**C**). Pre-term groups had <37 weeks of gestation: P (3–12 days postpartum), and PT (when gestational plus chronological age reached 37 or more weeks, corrected age). Full-term group (T) had ≥37 weeks of gestation.

The most abundant phylum was Firmicutes (85.6–80.5 relative abundance %), followed by Proteobacteria (10.2–13.4%), Actinobacteria (2.5–5.6%), and Bacteroidetes (0.6–1.4%) (Figure 1A). The core genera were *Staphylococcus* (13.60–36.89%) and *Streptococcus* (66.17–36.13%). Less abundant genera, such as *Pseudomonas* (2.67–0.57%), *Acinetobacter* (2.24–0.30%), *Corynebacterium* (1.22–0.53%), and *Cutibacterium* (1.18–0.33%) were found in all samples. Nearly all human milk samples contained *Rothia* (1.93–0.03%), except for one from the P group. The frequencies of *Lactobacillus* (0.76–0.26% abundance) and *Bifidobacterium* (2.71–0.01% abundance) were 85% and 55%, respectively. *Enterobacter*, *Klebsiella*, and *Gemella* occurred in at least 85% of the samples.

### 3.2. Changes over Chronological Age of Pre-Term Human Milk Microbiota (P vs. PT)

When pre-term groups (P and PT) were analyzed according to chronological age, no differences were observed at the phylum level. (Figure 1A). However, at the family level, an increase in *Carnobacteriaceae* (0.02–0.77%, *p* = 0.028) between groups P and PT was found (Figure 1B). Regarding the genera, the relative abundance of *Dolosigranulum* (0.01–0.52%, *p* = 0.043) and *Rothia* (0.03–0.76%, *p* = 0.021) increased from groups P to PT. *Enterobacter* diminished from 1.80 in group P to 0.06% in group PT (*p* = 0.028). Otherwise, *Lactobacillus* and *Veillonella* tended to increase from groups P to PT (0.27 vs. 0.74%, *p* = 0.090, and 0.51 vs. 2.69%, *p* = 0.070), but this trend was not significant (Figure 1C).

**Figure 2:**
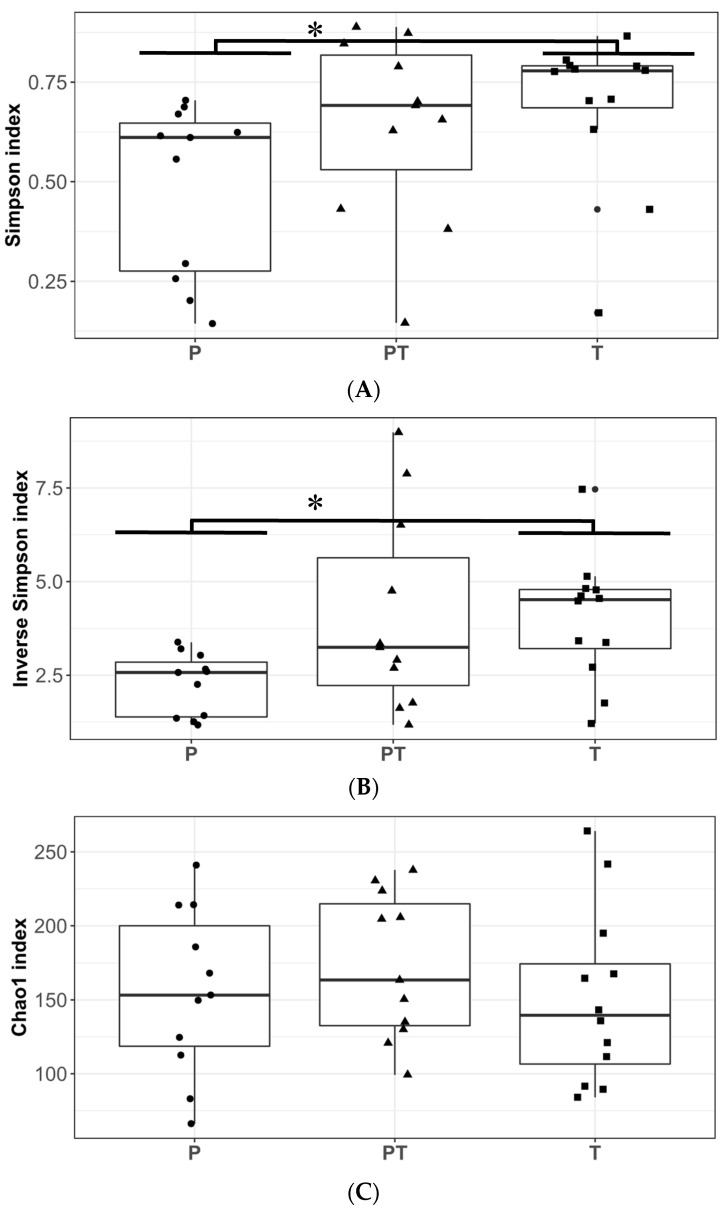
Alpha diversity and richness indices of human milk samples. (**A**) Simpson, (**B**) Inverse Simpson, and (**C**) Chao1 indices. Pre-term groups had <37 weeks of gestation: P (3–12 days postpartum), and PT (when gestational plus chronological age reached 37 or more weeks, corrected age). Full-term group (T) had ≥37 weeks of gestation. Mann–Whitney U test for independent non-parametric samples, * *p* < 0.05.

**Figure 3:**
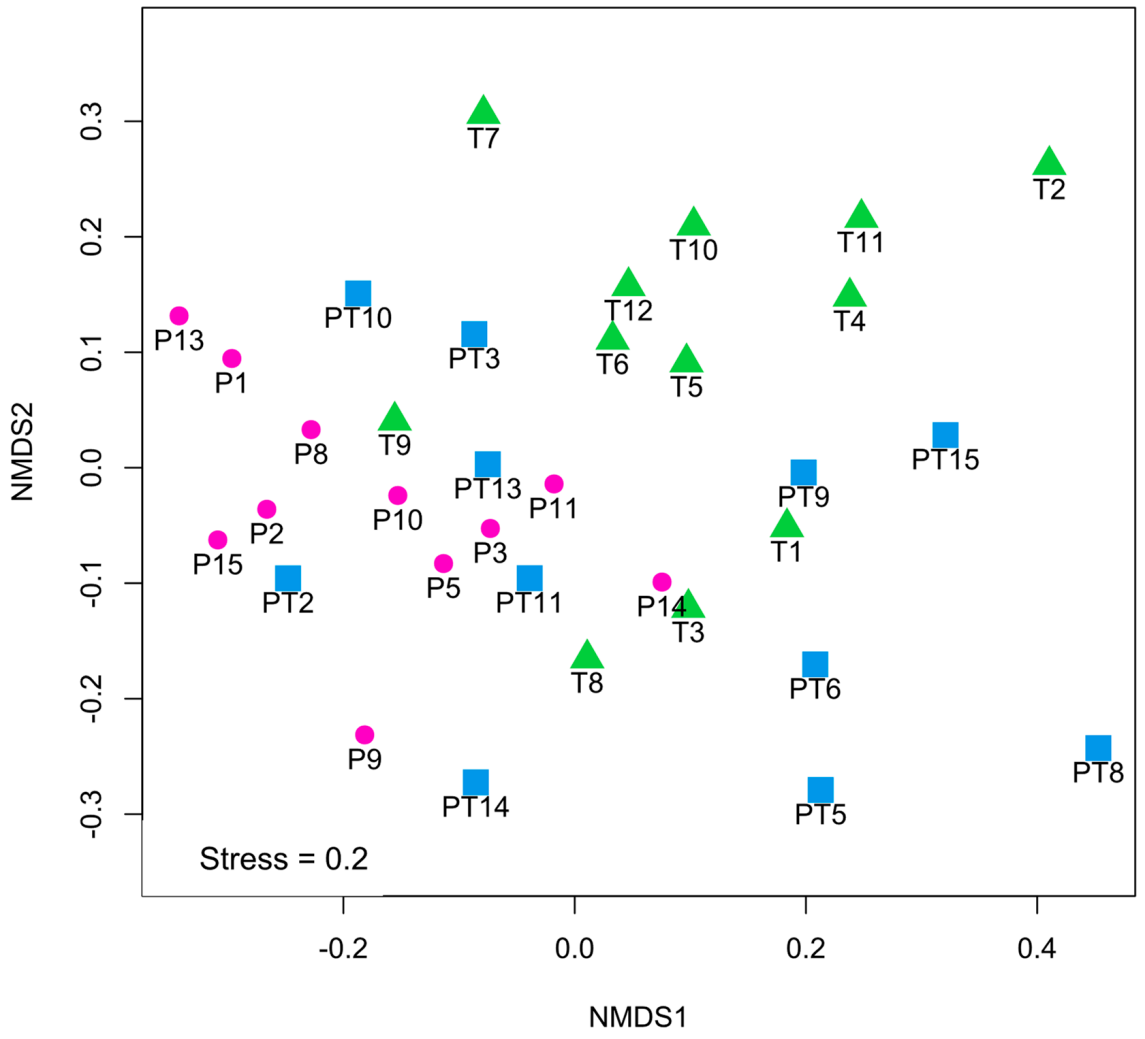
Non-metric multidimensional scaling (NMDS) plot corresponding to the human milk samples. Pre-term groups, <37 week of gestation: P (days 3–12 postpartum) and PT (when gestational plus chronological age reached 37 or more weeks, corrected age). Full-term group (T) had ≥37 weeks of gestation.

According to the alpha diversity indices, group PT was not different from group P (Figure 2). In the NMDS plot (Figure 3), there was a greater data dispersion of points in group PT, as compared to group P, showing a non-defined pattern. The PERMANOVA analysis did not show any changes between groups P and PT in the community structure.

### 3.3. Differences in Human Milk Microbiota Profiles in Mothers with Full-Term and Pre-Term Infants at Similar Corrected Ages (T vs. PT)

At the phylum level, no significant differences in the relative abundance were observed between groups PT and T (Figure 1A). When the family level was considered, group PT had a higher abundance, as compared to group T, of *Atopobiaceae* (0.29 vs. 0.00%, *p* = 0.013), *Veillonellaceae*, (2.70 vs. 0.43%, *p* = 0.044) and *Beijerinckiaceae* (0.29 vs. 0.09%, *p* = 0.016), respectively (Figure 1B). Conversely, as compared to the PT samples, group T presented a higher abundance of *Propionibacteriaceae* (0.75 vs. 0.33%, *p* = 0.019) and *Pseudomonadaceae* (2.71 vs. 0.56%, *p* = 0.037), respectively.

At the genus level, as compared to group PT, group T was richer in *Ralstonia* (4.49 vs. 0.00%, *p* = 0.006), *Cutibacterium* (0.75 vs. 0.33%, *p* = 0.016) and *Pseudomonas* (2.71 vs. 0.56%, *p* = 0.037), respectively, but had lower abundances of *Atopobium* than the PT samples (0.00 vs. 0.29%, *p* = 0.013). A trend towards a reduction in *Veillonella* content was observed in T samples, as compared to group PT, but it was not significant (2.69 vs. 0.37%, *p* = 0.051), respectively (Figure 1C). Furthermore, *Staphylococcus haemolyticus* presented a lower abundance in group T, as compared to group PT (0.00 vs. 0.08%, *p* = 0.004), respectively. There were no differences between groups PT and T in alpha diversity or richness (Figure 2). In addition, the PT samples presented a higher data dispersion, as compared to group T. The PERMANOVA analysis did not show any significant differences between groups PT and T.

### 3.4. Differences in Human Milk Microbiota Profiles in Mothers with Full-Term and Pre-Term Infants at the Same Chronological Ages (P vs. T)

At the phylum level, no differences were observed between groups P and T in relative abundance (Figure 1A). Instead, the T samples presented higher levels of *Streptococcaceae* (37.41 vs. 13.59%, *p* = 0.006) and *Micrococcaceae* (2.01 vs. 0.14%, *p* = 0.001), respectively, as compared to group P (Figure 1B). On the other hand, the families *Staphylococcaceae* (66.27 vs. 36.65%, *p* = 0.004) and *Rhodobacteraceae* (0.13 vs. 0.03%, *p* = 0.019) showed higher abundance in group P than in group T, respectively. In addition, *Staphylococcus* (66.26 vs. 36.65%, *p* = 0.004) and *Paracoccus* (0.10 vs. 0.01%, *p* = 0.044) presented higher levels in group P than group T, respectively (Figure 1C). However, higher abundances of *Streptococcus* (37.33 vs. 13.57%, *p* = 0.007), *Ralstonia* (4.49 vs. 0.00%, *p* = 0.006), *Rothia* (1.95 vs. 0.03%, *p* = 0.000), and *Stenotrophomonas* (0.15 vs. 0.00%, *p* = 0.037) were observed in group T, as compared to the P samples, respectively (Figure 1C).

Considering the abundance and significance of *Staphylococcus* in HM, we pursued the identification of potential species from this genus. We found *Staphylococcus aureus* (OTU_6), *Staphylococcus haemolyticus* (OTU_7, OTU_9, OTU_13, and OTU_23), and a non-cultivable species of *Staphylococcus* (OTU_1 and OTU_4) in the collected HM samples. A higher abundance of *S. aureus* was observed in group T than in group P (10.12 vs. 0.30%, *p* = 0.000), respectively, while *S. haemolyticus* (0.20 vs. 0.00%, *p* = 0.007) and the non-cultivable species (65.77 vs. 26.52%, *p* = 0.002) presented at higher levels in group P than in group T, respectively.

Higher alpha diversity indices were obtained for T samples, as compared to group P, when the Simpson (*p* = 0.004) and Simpson Inverse (*p* = 0.005) indices were considered, but the differences were not observed for richness according to the Chao1 index (*p* = 0.840) (Figure 2). In the NMDS (Figure 3), the samples from groups P and T were distributed to the left and to the right side of the plot, respectively. When the taxonomic compositions were analyzed by PERMANOVA, the samples from group P were significantly different from those from group T (*p* = 0.004). A SIMPER analysis determined the OTUs that were responsible for the observed differences. OTU_1, assigned as *Staphylococcus*, contributed up to 30% of the observed differences, while OTU_2 and OTU_3 contributed up to 54%, both identified as *Streptococcus*.

### 3.5. Human Milk Microbiota and Its Association with Gestational, Chronological, and Corrected Ages

Gestational age and neonatal weight differed among groups (Supplementary Table S2). The administration of antibiotics and corticoids was more frequent in group P than in group T. No significant association was observed for the delivery mode, the prenatal corticoids, or the antibiotics (Supplementary Figure S1).

**Figure 4:**
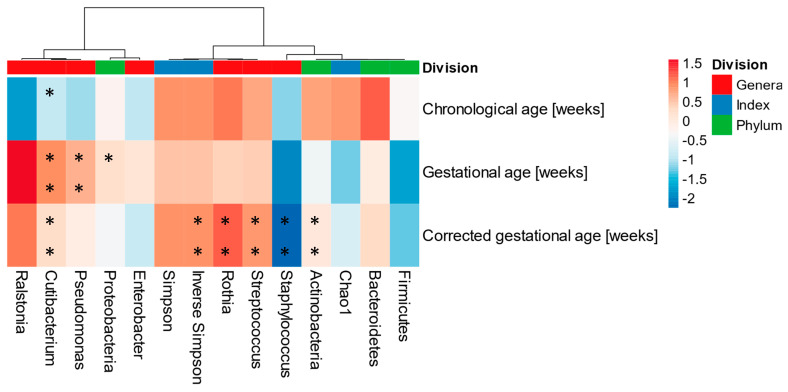
Human milk microbiota associated with gestational, chronological, and corrected ages (per weeks). Spearman’s rank correlation coefficient was performed on the taxa abundance at the phylum and genera (>1%) levels and alpha diversity index values. * *p* < 0.05, ** *p* < 0.001.

Spearman’s rank correlation coefficient determined the associations among chronological age, gestational age, and corrected age in HM microbiota. At the genus level, *Ralstonia*, *Rothia*, and *Streptococcus* were positively associated with corrected age and negatively correlated with *Staphylococcus* (Figure 4). A higher gestational age was associated with higher abundances of *Pseudomonas*, *Cutibacterium*, and *Ralstonia*. In addition, as the chronological age increased, *Ralstonia* appeared at lower levels. Furthermore, the corrected age was positively associated with the alpha diversity index in the pre-term birth group.

## 4. Discussion

HM plays a critical role in the development and well-being of infants, and its microbiota has been linked to beneficial effects during childhood since it provides most of the microbes needed to develop the infant microbiome and the modulation of the immune system [2]. It was thought that the origin of HM microbes was the entero-mammary pathway [24]. Microbes, which were sampled from the gut lumen by enteric dendritic cells, could have reached the mammary glands from the maternal gut by an endogenous route. Strict anaerobic genera, such as *Bifidobacterium*, *Clostridium*, and *Bacteroides* supported the theory of the entero-mammary pathway [1]. In addition, HM micro-organisms contribute around 25% of the infant’s gut microbiota [25]. It was known that correct gut colonization prevented necrotizing enterocolitis in pre-term infants, as well as later outcomes, such as food allergies [26], obesity, and other metabolic disorders [25]. In this study, we examined the characterization of bacterial communities in Argentinian HM and the differences between samples obtained from mothers that delivered pre-term and full-term infants. Furthermore, previous reports had not considered the corrected and chronological ages of pre-term infants in comparisons to the HM microbiota of full-term infants, thus these factors could contribute to novel approaches to address prematurity [24].

Maternal geographical location, diet, age, and chronological age have also been indicated to shape the HM microbiota composition [26,27]. Although the average of Proteobacteria and Bacteroidetes were conserved between Argentinian and Brazilian mothers, the Firmicutes phylum reached more than 80%, whereas the Actinobacteria phylum dropped to less than 6% in Argentinian HM, as compared to 70% and 14% in Brazilian HM [28]. Argentina and Brazil are neighboring countries in Latin America, but they have presented differences in microbiota, demonstrating the importance of geographical location, among other factors, which contribute to HM microbiota composition.

Our data were in agreement with previous studies that had identified a predominance of the genera *Staphylococcus* and *Streptococcus* in HM [29]. The maternal diet could modulate specific genera in HM. On the one hand, a diet rich in carbohydrates and low in proteins was positively correlated with higher levels of *Staphylococcus*. On the other hand, *Streptococcus* was directly associated with a higher intake of proteins and unsaturated fatty acids, as well as selenium and zinc. In addition, the genus *Bifidobacterium* and the former genus *Lactobacillus*, two genera recognized as beneficial microbes, were linked to unsaturated fatty acids and linoleic intake [5]. Half of the female Argentinian population has been classified as overweight or obese, and the typical daily diet includes a high intake of sugars, refined grains, white bread, rice, red meats, and saturated fats [30,31]. Moreover, it has been reported that HM from overweight or obese mothers presented higher levels of *Staphylococcus*, as compared to mothers with lower BMIs [32]. Almost 50% of the mothers in group P were classified as overweight or obese, as compared to less than 20% of the mothers in group T. This could explain the higher proportion of *Staphylococcus* in group P, as compared to group T, and might even explain the low levels of *Bifidobacterium* among the groups.

Gestational age could also contribute to changes in HM microbiota profiles, as evidenced by the low abundance of *Bifidobacterium* in the samples from pre-term deliveries, as compared to full-term deliveries [33]. In this study, groups P and T displayed different taxonomic compositions, whereas the PT samples presented a higher data dispersion. Alpha diversity was different between groups P and T according to the Simpson index. However, Urbaniak et al. (2016) did not observe differences in the alpha diversity in similar groups [34]. One limitation of our study was the relatively limited amount of HM samples analyzed, which could have biased the observed differences. In addition, the administration of antibiotics and the body mass index (BMI) >25 kg m^−2^ during pregnancy have been associated with lower diversity in HM in mothers with pre-term births [32].

*S. hemolyticus* was almost exclusively detected in group P. A high level of this species has been reported in HM from pre-term births, causing late-onset sepsis in pre-term neonates. Nevertheless, HM seems to be a rare source of *S. haemolyticus* [35]. Our results were also in agreement with those reported by Soerg et al. [34], who had found a higher abundance of *Staphylococcus* and, in particular, the species of *S. haemolyticus*, in mothers with pre-term infants than in those with full-term infants. Moreover, the gut and the skin of pre-term neonates were more commonly colonized with *S. haemolyticus* than full-term infants [36]. *S. aureus*, *Pseudomonas*, and *Ralstonia*, which were prevalent in group T, had also previously been found at high levels in HM from mothers with acute and subacute mastitis [37]. Considering that the majority of episodes of mastitis occur in the first six weeks after delivery, it would have been more likely to find these genera in groups T and P than in group PT. Recent studies have also revealed a loss of microbiome diversity in mastitis cases, but this was not observed in group T [38].

Regarding less abundant genera, such as *Veillonella* and *Rothia*, it has been reported that they were found in both the HM and in neonate’s feces from each mother–child pair, and thus, they must have been transferred from mother to child through breastfeeding [25]. In addition, according to the results obtained in this work, *Rothia* presented in higher abundance in group T than in group P, as had been previously observed in HM in several countries [33]. Some species of *Rothia* have also been postulated as a putative probiotic for gluten digestion due to its enzymatic production [39]. Otherwise, lower levels of *Veillonella* and *Rothia* in the infant gut have also been shown to enhance the risk of asthma in adulthood [40], and the consumption of insoluble fiber during lactation has been proposed as a strategy to increase the abundance of *Rothia* in HM [41]. 

When pre-term milk samples were analyzed at the corrected age sampling point, a reduction in the *Enterobacter* levels was observed, while an increase in *Dolosigranulum* was observed in group PT. The source of *Enterobacter* in HM could be the maternal areolar skin or infant oral sites in breastfeeding pairs [42]. Previous reports described a dominance of *Enterobacter* in the gut of pre-term infants, as this genus has been associated with a higher risk of developing necrotizing enterocolitis [43]. Moreover, *Dolosigranulum* and *Corynebacterium* were shown to be the predominant residents of the nasopharyngeal microbiota of breastfed infants, as compared to formula-fed infants, potentially exerting a protective effect against respiratory infections and wheezing in early infancy [44]. *Cutibacterium* and *Stenotrophomonas* were also reported in this study, but their impact on health is not fully understood [33].

From the colostrum to mature milk, HM changes its chemical and microbial composition. Several studies have suggested that, after the first 10 days of birth, there was an increase in alpha diversity [45], a decrease in *Staphylococcus*, and increases in *Bifidobacterium*, *Rothia*, *Veillonella*, *Granulicatella*, and *Lactobacillus* [33]. In this work, the full-term group presented a higher alpha diversity, a lower abundance of *Staphylococcus*, and a higher level of *Rothia*, as compared to group P. This could suggest that the human microbiota in pre-term deliveries differs from full-term deliveries, considering similar chronological ages. When groups T and PT were compared at their corrected ages, the diversity and some genera were similar, but this trend was not observed in all individual samples. In this sense, and according to the results obtained, the corrected age should be considered in pre-term births when analyzing HM microbiota maturation (Figure 4), as it is typically used when analyzing the overall growth and development of a child. Korp et al. (2018) also reported that postmenstrual age correlated more strongly with gut microbiota maturation than chronological age, in pre-term infants. Both the postmenstrual and corrected ages take into account the chronological-plus-gestational age [43]. This preliminary evidence suggested that HM and gut microbiota could be synchronized with the physical maturation and corrected age, in pre-term births.

The establishment of gut microbiota occurs early In life, and several perinatal factors shape it. HM is a source of beneficial micro-organisms, such as *Bifidobacterium* and *Lactobacillus* [46]. The predominance of the gut microbiota by *Bifidobacterium* has been considered an indicator of a healthy gut [43,46]. In this sense, interventions such as supplementation with bifidobacteria and bifidogenic components, including prebiotics, could be a path to promote the gut microbiota and the maturation of the gut-associated immune system in Argentinian mothers that deliver pre-term infants. Future studies in our region need to consider antibiotics administration, the BMI of the pregnant person, smoking habits, exclusive breastfeeding practices, as well as sample size, since these factors critically shape the microbiota in pre-term neonates [27,32].

We acknowledge that this study had some limitations, such as the relatively low number of samples and the lack of other longitudinal sampling points. These limitations could have influenced the variability of the observed data. Conducting this type of study in Argentina was novel, and some mothers were reluctant to provide milk samples, especially if several samples had been requested over time. In addition, the limited budget for research was also a condition that should be considered. However, for being the first of its kind, this study is valuable, and it establishes a foundation for future, more comprehensive studies.

## 5. Conclusions

This is the first report of the HM composition in lactating mothers in Argentina. Full-term delivery was associated with a higher diversity of microbes in HM than in pre-term delivery, and the ratio of *Streptococcus/Staphylococcus* was close to one. When HM samples were obtained for a second time in mothers that delivered pre-term children, no significant differences in the overall microbial profiles in HM were noted, as compared to full-term samples, suggesting that a microbial maturity could be achieved by supporting breastfeeding. The microbial composition of pre-term HM, at the corrected age, did not show significant differences, as compared to samples obtained from the full-term group. Corrected age is a variable commonly used in the public health sector when studying the overall development of children. Therefore, it would be appropriate to consider corrected age when studying the microbiota of HM in future studies.

## Supplementary Materials

The following supporting information can be downloaded at: https://www.mdpi.com/article/10.3390/microorganisms11041090/s1, Table S1. Regression coefficients and significances of clinical data association with milk microbiota at phylum, genera levels, and alpha diversity indices. Clinical data association with milk microbiota composition at phylum and alpha diversity indices. Delivery mode: Cesarean section/Vaginal. Prenatal administration of antibiotics and corticoids: Yes/No. β coefficients of univariate associations with linear regression are visualized for taxa abundance and alpha diversity index value. ** *p* < 0.05, ** *p* < 0.001. References [47,48,49,50,51] are cited in the supplementary materials.
