# Supplementary material for: Human Milk Microbiota Profile Affected by Prematurity in Argentinian Lactating Women
Source: Microorganisms. 2023 Apr 21;11(4):1090. doi: 10.3390/microorganisms11041090 (PMC10145235; doi:10.3390/microorganisms11041090)
Supplement: Supplementary file 1 [file microorganisms-11-01090-s001.zip › microorganisms-2259500-supplementary.pdf]

Supplementary methods

Data analysis

Raw sequences were processed using a modified version of the pipeline proposed by Logares [1] , described above. Reads were first analyzed for error correction using the algorithms based on Hamming graphs and Bayesian subclustering (BAYES HAMMER tool) [2] implemented in SPAdes v3.5.0 [3]. Then a quality check was performed using fastq_filter in USEARCH-v10 [4]. Reads that passed the quality control were analyzed using UNOISE2 [5] to define operational taxonomic units (OTUs) with no clustering (zero-radius OTUs [zOTUs]) [6]. The zOTU table was created with the function otutab in USEARCH-v10 [7]. OTUs identified as contaminants from the sequencing controls were also removed. Finally, taxonomy assignment of zOTUs was done by BLAST [8], using the SILVA database (SSU Ref 132 NR 99) as a reference. zOTUs ≤ 10 reads were removed.

References

1 . Logares R. ramalok/amplicon_processing: Workflow for Analysing MiSeq Amplicons based on Uparse. 2017 DOI: http://doi.org/10.5281/zenodo.259579

2 . Nikolenko SI, Korobeynikov AI, Alekseyev MA. BayesHammer: Bayesian clustering for error correction in single-cell sequencing. BMC Genomics. 2013;14(Suppl 1):S7.

3 . Nurk S, Bankevich A, Antipov D, et al. Assembling Single-Cell Genomes and Mini-Metagenomes From Chimeric MDA Products. Journal of Computational Biology. 2013 Oct;20(10):714–37.

4 . Edgar RC, Flyvbjerg H. Error filtering, pair assembly and error correction for next-generation sequencing reads. Bioinformatics. 2015 Nov;31(21):3476–82.

5 . Edgar RC. UNOISE2: improved error-correction for Illumina 16S and ITS amplicon sequencing. bioRxiv. 2016 DOI: https://doi.org/10.1101/081257

6 . Edgar RC. Updating the 97% identity threshold for 16S ribosomal RNA OTUs. Bioinformatics. 2018 Jul;34(14):2371–5.

7 . Edgar RC, Flyvbjerg H. Error filtering, pair assembly and error correction for next-generation sequencing reads. Bioinformatics. 2015 Nov;31(21):3476–82.

8 . Altschul SF, Gish W, Miller W, et al. Basic local alignment search tool. Journal of Molecular Biology. 1990 Oct;215(3):403–10.

Supplementary material.

Supplementary S1.

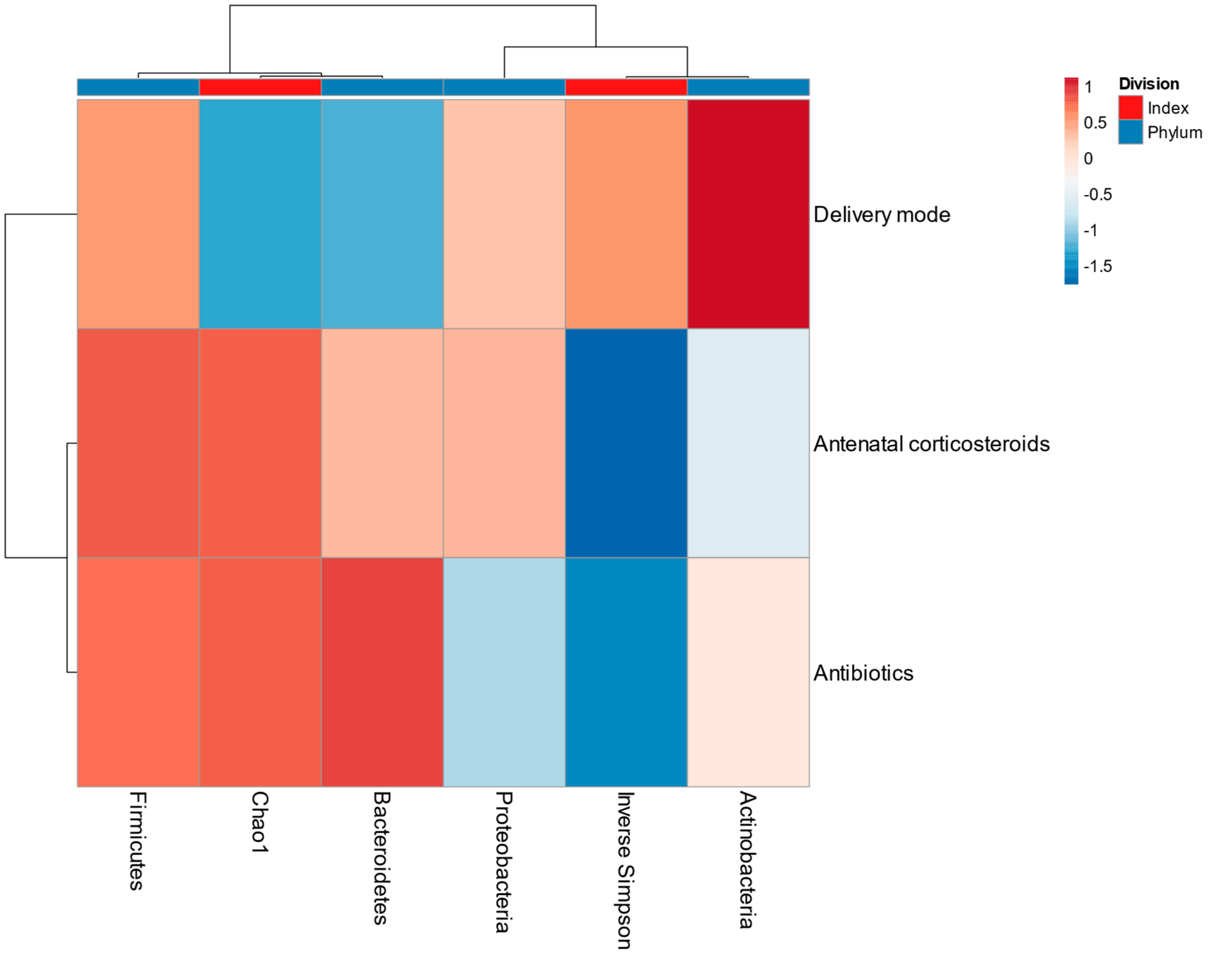

Figure S1. Clinical data association with milk microbiota composition at phylum and alpha diversity indices. Delivery mode: Cesarean section/Vaginal. Prenatal administration of antibiotics and corticoids: Yes/No. β coefficients of univariate associations with linear regression are visualized for taxa abundance and alpha diversity index value. $*p < 0.05$, $**p < 0.001$.

Table S1. Regression coefficients and significances of clinical data association with milk microbiota at phylum, genera levels, and alpha diversity indices.

	β	p value
Delivery mode *vs*. Actinobacteria phylum	0.124	0.484
Delivery mode *vs*. Bacteroidetes phylum	-0.154	0.384
Delivery mode *vs*. Firmicutes phylum	0.052	0.771
Delivery mode *vs*. Proteobacteria phylum	0.021	0.904
Delivery mode *vs*. Chao1 index	-0.167	0.351
Delivery mode *vs*. Inverse Simpson index	0.057	0.749
Delivery mode *vs*. Shannon index	-0.005	0.976
Antenatal Corticosteroids *vs*. Actinobacteria phylum	-0.079	0.659
Antenatal Corticosteroids *vs*. Bacteroidetes phylum	0.063	0.663
Antenatal Corticosteroids *vs*. Firmicutes phylum	0.137	0.441
Antenatal Corticosteroids *vs*. Proteobacteria phylum	0.065	0.716
Antenatal Corticosteroids *vs*. Chao1 index	0.133	0.455
Antenatal Corticosteroids *vs*. Inverse Simpson index	-0.259	0.125
Antenatal Corticosteroids *vs*. Shannon index	-0.064	0.718
Antibiotics *vs*. Actinobacteria phylum	-0.004	0.984
Antibiotics *vs*. Bacteroidetes phylum	0.171	0.334
Antibiotics *vs*. Firmicutes phylum	0.139	0.538
Antibiotics *vs*. Proteobacteria phylum	-0.154	0.383
Antibiotics *vs*. Chao1 index	0.152	0.391
Antibiotics *vs*. Inverse Simpson index	-0.262	0.134
Antibiotics *vs*. Shannon index	-0.121	0.495

Table S2. Clinical data of the mother-child pair

Parameter	Preterm	Term	P value
**Number of samples**	24	12	Not applicable
**Number of mothers**	12	12	Not applicable
**Days postpartum, 1st sample**	8 ± 3	8 ± 3	0,799[2]
**Days postpartum, 2nd sample**	22 ± 9	Not applicable	Not applicable
**Relation vaginal/Caesarean section**	3:09	4:08	Not applicable
**% Caesarean section**	75 (9:12)	67 (8:12)	Not applicable
**Gestational age (weeks)**	33,4 ± 1,2	38,3 ± 1,2	0,000[1]
**Neonatal weight (g) at birth**	1968 ± 308	2925 ±484	0,000[1]
**Neonate Sex (M/F)**	6:06	5:07	Not applicable
**Age (years) of mothers**	25,7 ± 7,5	29,1 ± 8,2	0,300[1]
**Allergies of mothers (Yes / No)**	5:07	4:07	Not applicable
**Diabetes before pregnancy (Yes / No)**	0:12	1:11	Not applicable
**Hypo / Hyper Thyroidism (Yes / No)**	2:10	1:11	Not applicable
**Anemia during pregnancy (Yes / No)**	2:10	2:10	Not applicable
**Gestational diabetes (Yes / No)**	2:10	2:10	Not applicable
**Antenatal corticosteroids (Yes / No)**	11:01	2:10	Not applicable
**Antibiotics (Yes / No)**	10:02	7:05	Not applicable
**Active smoker during pregnancy (Yes / No)**	2:10	2:10	Not applicable
**Passive smoker during pregnancy (Yes / No)**	5:07	3:09	Not applicable

[1] Student's T-test for independent samples for parametric data

[2] Mann-Whitney U test for non-parametric data

* $p < 0.05$, significant
